# Amyloid angiopathy of the floor of the mouth: a case report and review of the literature

**DOI:** 10.1186/1752-1947-1-117

**Published:** 2007-10-29

**Authors:** Daniel D Kokong, Titus S Ibekwe, Clement A Okolo, Aliyu M Kodiya, James A Fasunla, Onyekwere GB Nwaorgu, Effiong EU Akang

**Affiliations:** 1 Department of Otorhinolaryngology, University College Hospital, Ibadan, Nigeria; 2 Department of Pathology, University College Hospital, Ibadan, Nigeria

## Abstract

Amyloidosis is a rare disease characterised by the deposition of insoluble extracellular fibrillar proteins in various tissues of the body. The pattern of manifestation is organ dependent and also on whether the disease is localised or systemic, primary or secondary.

Though the disease is usually fatal with a 5-year survival rate of 20%, there is still paucity of literature on this disease entity worldwide. Diagnosis has remained mostly at autopsy.

A case of amyloid angiopathy involving the submandibular gland and floor of the mouth with an associated fatal bleed is reported. The purpose of this case report is to reiterate the importance of a high index of suspicion in the approach to the management of head and neck swellings.

## Introduction

Amyloidosis is a rare disease characterised by the deposition of an insoluble extracellular fibrillar protein in various tissues of the body. It can be primary systemic, secondary systemic, localised, myeloma-associated or hereditary-familial. These categories constitute 56%, 8%, 9%, 26% and 1% respectively [1]. The primary systemic form affects mainly mesenchymal tissues such as the heart, tongue, and gastro-intestinal tract (GIT). The secondary form is however associated with destructive chronic inflammatory diseases such as tuberculosis, leprosy, rheumathoid arthritis, ankylosing spondylitis and osteomyelitis. This form affects primarily the kidneys, adrenals, liver and spleen. About 12% of myeloma patients develop amyloidosis [2].

All types of amyloidosis consist of a major fibrillar protein derived from a precursor protein through conversion by cells suspected to be macrophages, though yet to be confirmed. These proteins define the type of amyloid in 95% of cases. In addition there are the minor fibrillar proteins that include a P-component derived from Serum Amyloid P-component (SAP), apolipoprotein E(apoE), heparan sulphate proteoglycan, glycosaminoglycans and some other glycoproteins which constitute the remaining 5%. About 20 different fibrils have been described in human amyloidosis, each with a different clinical picture.

The disease has been called different names by various authors including paramyloidosis, malignant amyloidosis, essential amyloidosis, Wilds disease, Wild-Lubarschs disease, systemic amyloidosis, and generalised amyloidosis amongst others, signifying the controversies surrounding the disease [3,4].

Literature on amyloid angiopathy globally is scanty and specific literature on the involvement of the submandibular salivary gland and the floor of the mouth is even rarer. It is the purpose of this communication to report a case of amyloid angiopathy involving the submandibular salivary gland and the floor of the mouth, the first report of its kind from the West African Sub-region. The problems with diagnosis and management are highlighted.

## Case presentation

A 56 year old retired male civil servant who had been referred from the surgical outpatient department with a Fine Needle Aspiration Cytology (FNAC) report of benign salivary gland tumour, presented to our ENT department in the same institution, with a one year history of gradual but progressive bilateral swellings of the submandibular region. There was no associated variation in size with feeding. It was initially painless but the patient had developed some burning sensation involving the head and neck region. There were no associated purulent discharge, halitosis, dryness in or of the mouth or eyes and no upper lid swelling. About the same time, he noticed progressive tongue enlargement that almost filled the oral cavity and resulted in affectation of his speech. There were no other laryngologic, rhinologic or otologic symptoms. His appetite was good with no weight loss or abdominal discomfort. He had no symptoms referable to the musculoskeletal system.

Physical examination revealed an anxious ill-looking middle-aged man with an open mouth posture and" hot potato" speech. In addition, he had thickened skin of the head and neck region and massive macroglossia obscuring the visualization of the oropharynx. The tongue appeared depapillated but moist and pink. There was bilateral submandibular salivary gland enlargement, each measuring 4 by 3 cm, bosselated, firm to hard in consistency and attached to skin. There was no lacrimal gland swelling or slanting of the palpebral fissures.

A provisional diagnosis of Multiple Myeloma with Amyloidosis was made with Mikulicz disease as differential diagnosis.

Investigations done included: Full blood count with PCV-38%, WBC-6,400/cmm; neutrophil-42% (low), lymphocytes-46% (slightly above normal), monocytes-7% (normal), platelets-123,000/cmm (low). Electrolytes, Urea and Creatinine were within normal limits while urinalysis, was normal. HIV 1 and 2 screening by the rapid kit method was non-reactive. An Ultrasound scan (USS) of the neck revealed a normal thyroid gland and diffuse enlargement of the submandibular glands devoid of stones, cysts, or lymph node enlargement.

He had an open biopsy of the submandibular gland in which a 2.0 by 0.5 centimeter wedge biopsy was taken from the left submandibular gland. This provoked a torrential bleed giving the impression of a major arterial injury but careful search revealed no identifiable blood vessel and the idea of external carotid artery ligation was jettisoned after having finally secured haemostasis by mass suturing (tissue was friable). Pressure dressing was also applied. About 250–500 mls of blood was lost and patient was subsequently admitted for observation.

He was discharged after 48 hours with a clinical diagnosis of submandibular salivary gland haemangioma. He bled spontaneously on the 25^th ^and 29^th ^postoperative days for which he was readmitted.

He was then planned for a CT-angiography while an Interventional Radiologist was consulted for a possible selective embolisation; the facility however was not available. Independent reviews by the Surgical Oncologist and the Cardiovascular Surgeon were not suggestive of the likelihood of a named vascular structure at the region in question. However the swelling had become massive almost compromising the airway, making neck exploration hazardous. Preliminary histology of the initial specimen revealed chronic inflammation with presence of thick walled vessels suspicious of amyloid angiopathy for which special stain with Congo red was done.

**Figure 1:**
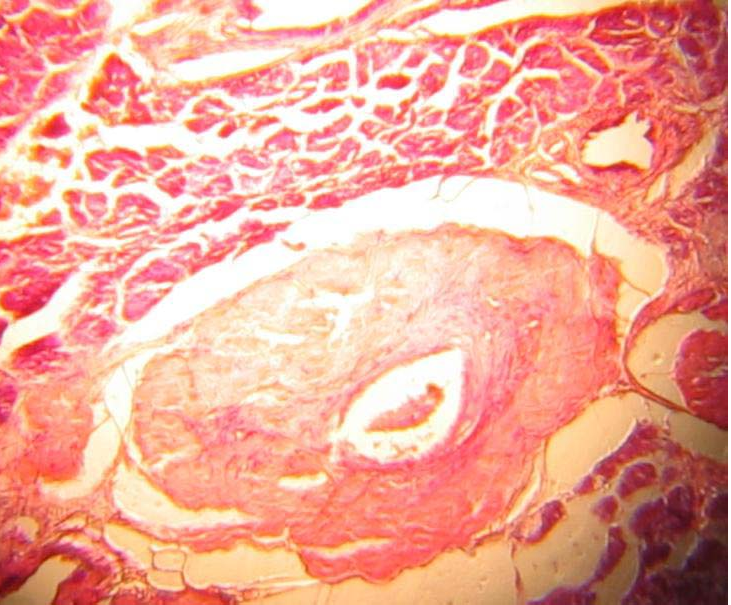
Photomicrograph of submandibular gland showing a large thick walled artery. H&E ×50.

Radiotherapist review recommended haemostatic dose of irradiation of 15GY in 3 fractions for a week. While awaiting radiotherapy and final histologic diagnosis, he had a fatal sudden and massive bleed, which defied all resuscitative measures. Histology confirmed amyloid angiopathy. Figure 1 is a photomicrograph of the amyloid angiopathy in the submandibular gland.

## Discussion

Rudolph Virchow in 1854, because of his interest in this disease which affected several body tissues and organs causing them to become waxy looking and show reaction with iodine and sulphuric acid, similar to that demonstrated by corpora amylacea of nervous tissue, concluded that the material did not have all the qualities of either starch or cellulose, but that it was probably isomeric for both. He then called it a name earlier used by botanists for starch-like substances-amyloid. He was also the first to describe vascular involvement of this disease.

The condition, however, that is known today as amyloidosis was first described by Rokitansky in 1842. It remains one of the challenging diseases facing the clinician, pathologist, and the chemist as its aetiology and pathogenesis have remained elusive and speculative and its management, controversial. To date, no single treatment has been shown to alter the underlying pathology or disease progression [5].

There have been several classifications of Amyloidosis by different researchers [5,6]. Currently, "Amyloidologists" classify amyloidosis based on its constituent chemical fibrils into categories such as Light Chain Amyloidosis (AL), Amyloid Associated Protein (AA) and Transthyretin Amyloidosis (ATTR) and not based on clinical syndromes. Because a given biochemical form of amyloid for example, AA, may be associated with amyloid deposition in diverse clinical settings, a clinical-biochemical classification has been suggested [7].

On clinical grounds, the systemic or generalised pattern is subclassified into primary and secondary. It is primary systemic amyloidosis when associated with some immunocyte dyscrasias. This is the most common form seen in 5–15% of multiple myeloma. The amyloid fibril protein is AL. It is characterised by multiple osteolytic lesions in the skeletal system. As our patient never presented with bone pains or other skeletal symptoms, skeletal survey for multiple myeloma was not done. However, one cannot rule out that posibility, especially, if it was subclinical. Furthermore, bone marrow biopsy was not done as the suspicion was not strong enough, moreso that the urinary Bence-Jones protein was negative. This appears an oversight for the very reason that serum Bence-Jones protein was not done. However, clinical picture of a burning sensation in the head and neck region indicating peripheral nerve involvement, thickened skin and macroglossia are some of the presentations of this form of amyloidosis along with heart, kidney and gastrointestinal tract involvement [8]. Secondary systemic amyloidosis is seen in patients with destructive chronic inflammatory diseases. The amyloid fibril proteins here are of the AA type, suggesting that this form of amyloidosis is related to recurrent bouts of inflammation that is also seen in heredofamilial amyloidosis. The gene implicated in this type of amyloidosis is called the Pyrin gene. It is responsible for regulating inflammation via neutrophil inhibition. In mutation of this gene, minor traumas unleash a vigorous tissue damaging effect [9].

Amyloidosis is an uncommon disease with poor records world wide. This was also observed in our departmental records where this happened to be the only recorded histologically confirmed case. This could have been responsible for the initial delay in our diagnosis. In the USA, there are 1,275–3,200 new cases of primary systemic amyloidosis annually. This may not be unconnected with their level of awareness regarding the disease including that of the technological advancement, which makes sophisticated investigative tools easily accessible and affordable.

Generally, peak age incidence is in the fifties, as was in our case, with a male: female ratio of 2:1 and no racial bias. The disease is rapidly fatal within 1 – 3 years of diagnosis. Diagnosis is mostly made at post mortem, from renal, cardiac and central nervous system involvement. The 5-year survival rate has been put at 20% [5,7,10].

Diagnosis of amyloidosis is anchored on high index of suspicion with detailed clinical history, thorough physical examination and comprehensive investigations either to confirm or rule out the presence of the disease. The haemogram of this patient showed a normal red cell count and packed cell volume. Though the WBC was within normal range, the differential showed a reversal of the neutrophil and lymphocyte count ratio, with the former lower than normal. This could be a technical or observer error. The platelet count was lower than normal and could have contributed to the bleeding or compounded an already existing disease or a manifestation of marrow failure seen in myelomatosis! The latter appears not a possibility as the haemogram was normal while the former could be an isolated finding.

Bone marrow biopsy, erythrocyte sedimentation rate, serum electrophoresis for monoclonal immunoglobulin light chains as well as electron microscopy, X-ray crystallography and infra-red spectroscopy that demonstrates the B-pleated sheet conformation, responsible for the green birefringence, could have resolved this dilemma. A point to note here is that a great majority of patients with AL amyloid do not have the classic multiple myeloma or any other overt B-cell neoplasm but virtually all have monoclonal immunoglobulins or free light chain or both in their serum, urine or both.

Another investigative tool of importance could have been radionuclide scan using radiolabelled serum amyloid P-component (SAP) [11,12]. Serum biochemistry was normal signifying satisfactory functional kidneys and FNAC was misleading, confirming the limited role it plays in investigating swellings.

Though Congo red staining result came out late it still gave us a confirmation of the diagnosis which would go a long way at improving our management protocol.

**Figure 2:**
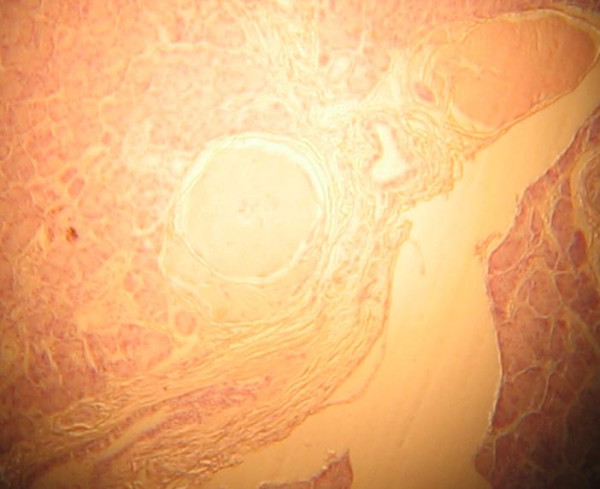
Congo red staining showing salmon pink appearance of thickened arterial wall consistent with amyloid deposits. ×50.

## Conclusion

Amyloidosis, though a rare disease is not uncommon. The need for a detailed and comprehensive patient evaluation cannot be overemphasized. So also is the role of a high index of suspicion in evaluating patients with head and neck swellings.

## Competing interests

The author(s) declare that they have no competing interests.

## Authors' contributions

DDK was the principal investigator and the conceiver of the idea for publication. TSI performed the literature search and prepared the manuscript. CAO prepared the slides and the microfilms of the specimen. AMK assisted in the surgery and management of the patient. JAF assisted in the surgery and literature search. OGN was the principal surgeon and revised the manuscript. EUA read the slides and reviewed the manuscripts.
